# Effects of Vector Maturation Time on the Dynamics of Cassava Mosaic Disease

**DOI:** 10.1007/s11538-021-00921-4

**Published:** 2021-06-28

**Authors:** F. Al Basir, Y. N. Kyrychko, K. B. Blyuss, S. Ray

**Affiliations:** 1 Department of Mathematics, Asansol Girls’ College, Asansol, West Bengal 713304 India; 2 Department of Mathematics, University of Sussex, Falmer, Brighton, BN1 9QH UK; 3 Systems Ecology and Ecological Modeling Laboratory, Department of Zoology, Visva-Bharati, Santiniketan, 731235 India

**Keywords:** Whitefly vector, Maturation delay, Plant viral disease, Hopf bifurcation, Numerical stability analysis

## Abstract

Many plant diseases are caused by plant viruses that are often transmitted to plants by vectors. For instance, the cassava mosaic disease, which is spread by whiteflies, has a significant negative effect on plant growth and development. Since only mature whiteflies can contribute to the spread of the cassava mosaic virus, and the maturation time is non-negligible compared to whitefly lifetime, it is important to consider the effects this maturation time can have on the dynamics. In this paper, we propose a mathematical model for dynamics of cassava mosaic disease that includes immature and mature vectors and explicitly includes a time delay representing vector maturation time. A special feature of our plant epidemic model is that vector recruitment is negatively related to the delayed ratio between vector density and plant density. We identify conditions of biological feasibility and stability of different steady states in terms of system parameters and the time delay. Numerical stability analyses and simulations are performed to explore the role of various parameters, and to illustrate the behaviour of the model in different dynamical regimes. We show that the maturation delay may stabilise epidemiological dynamics that would otherwise be cyclic.

## Introduction

One of the major challenges to successful agriculture comes from plant viruses that target grains, legumes and vegetables, resulting in significant economic losses (Sanfaçon 2017; Kumar et al. 2020). With plants being immobile, plant viruses are primarily vectored into them by arthropods, and more specifically, hemipteran insects (Perring et al. 1999; Whitfield et al. 2015), including aphids, leafhoppers and whiteflies (Perring et al. 1999; Jeger et al. 2004). One example of a notable viral disease of plants is the so-called *cassava mosaic disease*, characterised by distortion and mottling of plant leaves, chlorosis, intraveinal netting and stunting of affected plants (Legg 2008; Tompkins 1937; Sseruwagi et al. 2004), which often results in them producing virtually no yield. Similar diseases, such as the *Jatropha curcas* mosaic disease, affect a variety of important agricultural crops (Kumar et al. 2020).

Plant viruses are characterised by a significant diversity of lifestyle (Roossinck 2013), which affects the types of vectors that can carry them, and the interactions between these vectors and plants. One convenient classification of plant viruses is based on the so-called *persistency* (Perring et al. 1999; Roossinck 2013). Non-persistent plant viruses are characterised by a rapid acquisition of virus from plants by vectors through simple probing, after which the vectors can move to other plants (Roossinck 2013). In contrast, persistent plant viruses are usually processed through their insect vectors’ gut, and then, the insect will normally remain viruliferous for the duration of their lifetime. Such viruses require a stronger association between vectors and plants to ensure adequate virus acquisition through feeding (Mauck et al. 2012).

In this paper, we focus on the cassava mosaic disease, caused by members of the *Begomovirus* family, such as African Cassava Mosaic Virus (Fauquet and Fargette 1990) or Indian Cassava Mosaic Virus (Saunders et al. 2002), and transmitted by whiteflies (*Bemisia tabaci*) (Dubern 1994). Begomoviruses are persistently transmitted viruses (Liu et al. 2013; Moreno-Delafuente et al. 2013), and once the vector acquires such virus through feeding on infected plant, the virus then spreads to their midgut and then to their salivary glands, at which point they are able to pass it on to uninfected plants during feeding on those plants (Brown and Czosnek 2002). Importantly, at the pupal stage, whiteflies do not feed on plants, and thus, the virus can only be acquired and transmitted by adult whiteflies. Bearing in mind this observation together with the fact that maturation period of a whitefly is between twelve and twenty days in temperate climate (Jones 2003), it is important to include maturation period explicitly in the dynamics for transmission of plant viruses between plants and disease vectors.

In terms of mathematical modelling, a large number of models have looked into dynamics of interactions between hosts and their parasites. In the context of population ecology, starting with the pioneering work of Anderson and May (1978a, 1978b) and May et al. (1981), there has been a strong interest in understanding the role of host density dependence in determining the dynamics of parasites, and similarly, the role of prey density dependence in predator–prey interactions. Whereas early models focused on instantaneous interactions between prey and predator, or between host and parasite, to achieve better biological realism, a number of subsequent models also analysed the effects of various time delays. In the framework of age- and/or stage-structured populations, a large number of papers have considered the role of maturation delay for single species (Aiello and Freedman 1990; Aiello et al. 1992; Cooke et al. 1999a; Kuang 1993), as well as for multi-species problems, such as predator–prey interactions (Gourley and Kuang 2004; Banerjee and Takeuchi 2017), as well as distributed delays and spatio-temporal population dynamics (Al-Omari and Gourley 2005a, b). A number of papers have investigated the effects of vector maturation delay in the context of vector-borne animal diseases (Cooke et al. 1999b; Fan et al. 2010; Ngwa et al. 2010; Martcheva and Prosper 2013). These papers showed that maturation delays can destabilise vector dynamics and generate periodic solutions through a Hopf bifurcation, as well as more complex dynamics and chaos. Whereas in many of these and similar models, discrete time delays often result in the destabilisation of co-existence equilibria, Banerjee and Takeuchi (2017) have discussed the importance of biological considerations and justification for an appropriate inclusion of maturation time delay to avoid misleading conclusions, and they have also demonstrated that maturation delays can play a stabilising role.

In the context of modelling plant diseases, following an early work of Van der Plank (1963), who was the first to incorporate time delays in a model of plant epidemic to represent latent and infectious periods in the infected host tissue, a number of papers have subsequently looked at plant–vector interactions and transmission of plant viruses. Cunniffe et al. (2012) have explored the role of distribution of latent and infectious periods in compartmental models of plant disease, using a linear chain trick to represent gamma-distributed delays as multiple stages of infection. Buonomo and Cerasuolo (2015) studied plant–pathogen interactions with time delay representing the time it takes for a free-living inoculum to become infectious. Jackson and Chen-Charpentier (2018, 2017) have recently considered the effects of time delays representing incubation of virus in plants and vectors, focusing on numerical bifurcation analysis and identifying regions of stability of different steady states. Li et al. (2018) have subsequently complemented this analysis by analytical results based on centre manifold theory, which allowed them to identify boundaries of Hopf bifurcation of the positive equilibrium depending on time delays. Ray and Al Basir (2020) have considered a similar problem focusing only on within-plant incubation period. For within-plant dynamics, Neofytou, et al. (2016a, 2016b) have considered the dynamics of plant immune response to virus infections, and the role of time delays associated with plant maturation time and the propagation of a signal associated with RNA interference (RNAi) to different cells within a plant. From the perspective of control of vector-borne plant infections, several recent papers have looked at the effects of time-delayed interventions, such as roguing (i.e. removal of infected plants) or using insecticides, in response to awareness (Al Basir and Roy 2019; Al Basir et al. 2018a, b, 2019; Blyuss et al. 2020).

In this paper, we consider a model of cassava mosaic disease, with an emphasis on investigating the effects of whitefly maturation time on the dynamics of whitefly-borne plant infection. In the next section, we derive the model and establish its well-posedness. Section 3 is devoted to identifying different equilibria of the model and deriving conditions for their biological feasibility and stability in terms of system parameters and the maturation delay. In Sect. 4, numerical stability analysis and simulations are performed to illustrate various stability regions and different types of behaviour that are exhibited by the model. The paper concludes in Sect. 5 with a discussion of results and open problems.

## Model Derivation

To model the dynamics of transmission of cassava mosaic virus between plants and their vectors, following the methodology of Holt et al. (1997) we consider the populations of plants and whitefly vectors. Let *x*(*t*) and *y*(*t*) denote the numbers of healthy and infected plant, respectively. These can also be interpreted as densities when the dynamics is considered on some closed field or plantation. In the absence of disease vectors, it is assumed that the healthy plants exhibit logistic growth behaviour, with a linear growth rate *r* and a carrying capacity *K* (Venturino et al. 2016). The plants die or are harvested/removed at rate $\mu$. For the vector population, it is assumed that both healthy and infected plants serve as hosts for vector reproduction. The model of Holt et al. (1997) represents the growth of uninfected vector population in the following form (here, *u*(*t*) and *v*(*t*) denote populations of susceptible and infected vectors):

1 $$\begin{aligned} b(u+v)\left( 1-\frac{u+v}{a(x+y)}\right) , \end{aligned}$$

which describes the logistic growth of vectors, with the carrying capacity being proportional to the total number of plants. To account for the fact that whiteflies have a maturation time, which is non-negligible compared to their overall average life expectancy, we divide the vector population into immature (larva stage) $v_1(t)$ and mature (adult) vectors. Mature vectors are further subdivided into susceptible $v_2(t)$ and infected $v_3(t)$ populations. We assume that both uninfected and infected vectors can produce larvae, which then become pupae, and after a certain maturation period $\tau$, reach an adult stage.

Considering larval and pupal stages implicitly, the equation for susceptible mature vectors can be written as a possible modification of the above-mentioned vector growth term (1) that incorporates maturation time delay in the form of the delayed logistic growth

2 $$\begin{aligned} \frac{\mathrm{d}v_2}{\mathrm{d}t}=b\Big ( v_2 (t-\tau )+v_3(t-\tau )\Big )\left[ 1-\frac{v_2 (t-\tau )+v_3(t-\tau )}{a(x(t-\tau ) +y (t-\tau ))}\right] e^{-c\tau }-cv_2(t),\nonumber \\ \end{aligned}$$

where *c* is the death rate of vectors, and $e^{-c\tau }$ represents the probability of survival of immature vectors through the period of maturation $[t-\tau ,t]$. In contrast to Beddington and May (1975) and Taylor and Sokal (1976), in Eq. (2) the delayed host population contributes to fecundity of the vector population through ratio dependence (vector density relative to plant host density). In the context of mosquito-borne human diseases, such as malaria, Ngwa (2006) used a similar logistic-type delayed equation to model the dynamics of malaria, where human host population was considered to be a constant parameter, and the growth rate in the number of adult mosquito vectors was proportional to a time-delayed term representing the number of eggs laid by fertilised vectors some time ago and then going through the maturation period covering the stages of eggs, larva and pupa. Ngwa et al. (2010) noted a particular problem with the logistic growth term in that in order for the recruitment term to be positive, the number of vectors cannot exceed the carrying capacity, and this problem also applies to Eq. (2). As an alternative to logistic growth, a number of authors have represented recruitment of vectors using a Ricker function (Cooke et al. 1999b; Fan et al. 2010; Ngwa et al. 2010; Martcheva and Prosper 2013; Nie and Xue 2017), which has the functional form $\displaystyle {v\exp (-av)}$. Unlike those earlier models that considered density dependence in the birth term for vectors, in the context of modelling plant disease, we rather replace Eq. (2) by a Ricker-like term with ratio dependence

$$\begin{aligned} \frac{\mathrm{d}v_2}{\mathrm{d}t}=b \Big (v_2(t-\tau )+v_3(t-\tau )\Big )\exp \left[ -\frac{v_2(t-\tau )+v_3(t-\tau )}{a(x(t-\tau )+y(t-\tau ))}\right] e^{-c\tau }-cv_2. \end{aligned}$$

Virus transmission can only occur between an infected mature vector and a healthy plant, or between an infected plant and a healthy mature vector. Transmission of the virus between vectors and vertically in the vector is not possible in the model, and once a vector becomes infected, it will stay infected for the remaining duration of its lifespan. The rate of disease transmission between infected vectors and healthy plants is denoted by $\lambda$, and an equivalent rate of transmission from infected plants to healthy mature vectors is denoted by $\beta$. Since host density is not constant, to account for frequency-dependent disease transmission (Keeling and Rohani 2011; Wonham et al. 2006; Ross 1911), the transmission terms are thus taken to be $\lambda xv_3/(x+y)$ for disease transmission from infected vectors to susceptible plants, and $\beta yv_2/(x+y)$ for disease transmission from infected plants to susceptible mature vectors.

With the above assumptions, the model for the dynamics of mosaic disease takes the following form

3 $$\begin{aligned}&\displaystyle {\frac{\mathrm{d}x}{\mathrm{d}t}= rx\left[ 1-\frac{x+y}{k}\right] -\frac{\lambda xv_3}{x+y}-\mu x,}\nonumber \\&\displaystyle {\frac{\mathrm{d}y}{\mathrm{d}t}=\frac{\lambda xv_3}{x+y}-(\mu +\alpha )y,}\nonumber \\&\displaystyle {\frac{\mathrm{d}v_2}{\mathrm{d}t}=b \Big (v_2(t-\tau )+v_3(t-\tau )\Big )\exp \left[ -\frac{v_2(t-\tau )+v_3(t-\tau )}{a(x(t-\tau )+y(t-\tau ))}\right] e^{-c\tau }- \frac{\beta v_2 y}{x+y}-cv_2,}\nonumber \\&\displaystyle {\frac{\mathrm{d}v_3}{\mathrm{d}t}=\frac{\beta v_2 y}{x+y}-cv_3.} \end{aligned}$$

This system of equations has to be augmented by biologically appropriate initial conditions, which are taken to be as follows. Let *C* denote the Banach space of continuous functions $\phi : [-\tau , 0] \rightarrow {\mathbb {R}}^4_+$ equipped with the supremum norm,

$$\begin{aligned} \Vert \phi \Vert = \underset{-\tau \le \gamma \le 0}{\sup }\{|\phi _ 1(\gamma )|, |\phi _ 2(\gamma )|, |\phi _3(\gamma )|,|\phi _4(\gamma )|\}, \end{aligned}$$

where $\phi = (\phi _ 1, \phi _2, \phi _3,\phi _4)\in C([-\tau ,0],{\mathbb {R}}^4_+)$. In order to be biologically feasible, populations have to have nonnegative values, and thus, the initial condition for model (3) is taken in the form

4 $$\begin{aligned}&x(\gamma ) = \phi _1(\gamma ), ~y(\gamma ) = \phi _2(\gamma ),~v_2(\gamma ) = \phi _3(\gamma ),~~v_3(\gamma ) = \phi _4(\gamma ), \nonumber \\&\phi _{i}(\gamma )\ge 0, ~\gamma \in [-\tau , 0], ~i = 1, 2, 3, 4,\qquad \phi _{1,3,4}(0)>0. \end{aligned}$$

Biologically, this initial condition means that at time $t=0$, at least some number of vectors are already infected. While this condition may be quite natural for the vectors, as without them carrying the disease, the model would have no sense, and for plants, this also quickly results in the onset of infection, once the vectors start transmitting the virus to plants. It can be easily shown that the solution $(x(t), y(t), v_2(t), v_3(t))^T$ of model (3) with the initial condition (4) exists and is unique on $[0,+\infty )$ (Kuang 1993; Hale 1977). Furthermore, using the results of (Bodnar 2000; Yang et al. 1996), it is straightforward to show that solutions also remain nonnegative for all $t > 0$. Adding the first two equations of the system (3), we have

$$\begin{aligned} \frac{\mathrm{d}(x+y)}{\mathrm{d}t}= & {} rx\left[ 1-\frac{x+y}{k}\right] -\mu (x+y)-\alpha y\\\le & {} r(x+y)\left[ 1-\frac{x+y}{k}\right] -\mu (x+y). \end{aligned}$$

Since the function $rz(1-z/k)$ for $z\ge 0$ has the maximum value of *rk*/4, which is reached at $z=k/2$, we can rewrite last inequality as

$$\begin{aligned} \frac{\mathrm{d}(x+y)}{\mathrm{d}t}\le \frac{rk}{4}-\mu (x+y), \end{aligned}$$

which implies

$$\begin{aligned} \lim \sup _{t\rightarrow \infty }(x+y)\le M, \quad M=\max \left\{ \frac{rk}{4\mu },x(0)+y(0)\right\} . \end{aligned}$$

Similarly, using the fact that $x(t)+y(t)\le M$, we can add the last two equations of model (3) to find

$$\begin{aligned}&\displaystyle {\frac{\mathrm{d}(v_2+v_3)}{\mathrm{d}t}=b \Big (v_2(t-\tau )+v_3(t-\tau )\Big )\exp \left[ -\frac{v_2(t-\tau )+v_3(t-\tau )}{a(x(t-\tau )+y(t-\tau ))}\right] e^{-c\tau }}\\&\displaystyle {-c(v_2+v_3)\le b \Big (v_2(t-\tau )+v_3(t-\tau )\Big )\exp \left[ -\frac{v_2(t-\tau )+v_3(t-\tau )}{aM}\right] e^{-c\tau }}\\&\displaystyle {-c(v_2+v_3).} \end{aligned}$$

Since the function $bze^{(-z/aM)}e^{-c\tau }$ reaches its maximum $abMe^{-c\tau -1}$ at $z=aM$, we have

$$\begin{aligned} \displaystyle {\frac{\mathrm{d}(v_2+v_3)}{\mathrm{d}t}\le abMe^{-c\tau -1}-c(v_2+v_3),} \end{aligned}$$

which gives

$$\begin{aligned} \lim \sup _{t\rightarrow \infty }(v_2+v_3)\le N, \quad N=\max \left\{ \frac{abMe^{-c\tau -1}}{c},v_2(0)+v_3(0)\right\} . \end{aligned}$$

Hence, the region

$$\begin{aligned} {\mathbb {B}} = \left\{ (x, y,v_2,v_3) \in C([-\tau ,0], R^4_+) : 0\le x+y \le M,~0\le v_2+v_3\le N \right\} \end{aligned}$$

is positively invariant and attractive for all solutions in the positive octant.

## Equilibria and Their Stability

Model (3) has the following steady states: a plant-only *axial equilibrium* $E_1=\left( \frac{ k (r-\mu )}{r}, 0, 0, 0\right)$ characterised by the absence of vector population and the presence of only healthy plants, which exists, provided $r>\mu$, a *disease-free* steady state $E_2\left( {\bar{x}}, 0, {\bar{v}}_{2}, 0\right)$ with

$$\begin{aligned} \displaystyle {{\bar{x}}= \frac{k(r-\mu )}{r},\qquad {\bar{v}}_{2}= \frac{ak (r-\mu )\ln \left( {\bar{b}}/c\right) }{r},\qquad {\bar{b}}=b e^{-c\tau },} \end{aligned}$$

which exists for $r>\mu$ and ${\bar{b}}>c$, and the *endemic equilibrium* $E^*(x^*, y^*,v_2^*,v_3^*)$ with

5 $$\begin{aligned}&\displaystyle {x^*=-\frac{(\mu +\alpha )kB_1}{\beta r B_2},\qquad y^*=\frac{kB_1B_3}{\beta (\beta +c)rB_2},}\nonumber \\&\displaystyle {v^*_2=-\frac{kcB_1B_2}{(\beta +c)^2\lambda r},\qquad v^*_3=-\frac{kB_1B_3}{(\beta +c)^2\lambda r},} \end{aligned}$$

where

6 $$\begin{aligned}&\displaystyle {B_1=-a\beta \lambda \ln \left( {\bar{b}}/c\right) +c(r+\alpha )+\beta (r-\mu ),} \nonumber \\&\displaystyle {B_2=-a\lambda \ln \left( {\bar{b}}/c\right) -(\alpha +\mu ),} \displaystyle {B_3=-a\beta \lambda \ln \left( {\bar{b}}/c\right) +c(\alpha +\mu ).} \end{aligned}$$

From expressions for $x^*$ and $v^*_2$, it follows that $B_1$ and $B_2$ have to be of the opposite signs, and similarly, from the expression for $v^*_3$, we observe that $B_1$ and $B_3$ have to be of the opposite signs to ensure biological feasibility of the steady state $E^*$. The expression for $y^*$ shows that the only possibility how the endemic equilibrium $E^*$ can be biologically feasible is when $B_1>0$, $B_2<0$ and $B_3<0$. From the definitions of $B_2$ and $B_3$ in (6), it immediately follows that whenever $B_3<0$, the condition $B_2<0$ will also be automatically satisfied; hence, it is sufficient to require $B_2<0$, which, together with the condition $B_1>0$, can be recast as

7 $$\begin{aligned} \frac{c(\alpha +\mu )}{a\beta \ln \left( {\bar{b}}/c\right) }<\lambda <\frac{c(\alpha +\mu )+(\beta +c)(r-\mu )}{a\beta \ln \left( {\bar{b}}/c\right) }. \end{aligned}$$

If we define the *basic reproduction number* as

8 $$\begin{aligned} R_0=\frac{\beta \lambda {\bar{v}}_{2}}{c{\bar{x}}(\alpha +\mu )}=\frac{\beta \lambda a\ln \left( {\bar{b}}/c\right) }{c(\alpha +\mu )}, \end{aligned}$$

then the condition for existence of endemic equilibrium can be rewritten as follows

9 $$\begin{aligned} 1<R_0<1+\frac{(\beta +c)(r-\mu )}{c(\alpha +\mu )}. \end{aligned}$$

The first part of this inequality ensures that the rate of disease transmission is sufficiently high to make the disease endemic. The second part of this inequality is related to the fact that in our model, the population of hosts is reproducing logistically, but with only uninfected plants positively contributing to the growth, and hence, if the disease transmission rate is too high, then it can lead to extinction of plants. Since fecundity of vectors depends on host (plant) frequency, this will then lead to the extinction of vectors as well. Such scenario is familiar from other models of frequency-dependent transmission, in which a certain constraint between transmission and virulence should be satisfied to avoid parasite-driven host extinction (Boots and Sasaki 2003; Ryder et al. 2007). Such a scenario can take place even when viral disease does not result in plant death ($\alpha =0$), because if the transmission rate $\lambda$ is too high, new plants will not be replenished fast enough, and hence, all plants will become infected, at which point they will stop producing new healthy plants (because they are only produced by uninfected plants), and the extinction of both plant and vector populations will occur.

Linearisation near any equilibrium ${\widetilde{E}}({\widetilde{x}},{\widetilde{y}},{\widetilde{v}}_2,{\widetilde{v}}_3)$ yields the characteristic equation

10 $$\begin{aligned} \varDelta (\xi ) =\mid {\xi }{I}-\mathbf{A}-e^{-{\xi }{\tau }}{} \mathbf{B}\mid =0, \end{aligned}$$

where $\mathbf{A}=[A_{ij}]$ and $\mathbf{B}=[b_{ij}]$ are the following $4\times 4$ matrices,

$$\begin{aligned} \mathbf{A}=[a_{ij}]=\left[ \begin{array}{cccc} a_{11} &{}~~~~\displaystyle {-\frac{r{\widetilde{x}}}{K}+\frac{\lambda {\widetilde{x}}{\widetilde{v}}_3}{({\widetilde{x}}+{\widetilde{y}})^2}}&{} ~~~0 &{}~~\displaystyle {-\frac{\lambda {\widetilde{x}}}{{\widetilde{x}}+{\widetilde{y}}}}\\ \ \\ \displaystyle {\frac{\lambda {\widetilde{y}}{\widetilde{v}}_3}{({\widetilde{x}}+{\widetilde{y}})^2}}&{}~~~\displaystyle {-\frac{\lambda {\widetilde{x}}{\widetilde{v}}_3}{({\widetilde{x}}+{\widetilde{y}})^2}-(\alpha +\mu )}&{}~~~0 &{}~~\displaystyle {\frac{\lambda {\widetilde{x}}}{{\widetilde{x}}+{\widetilde{y}}}}\\ \ \\ \displaystyle {\frac{\beta {\widetilde{v}}_2{\widetilde{y}}}{({\widetilde{x}}+{\widetilde{y}})^2}}&{}~~~\displaystyle {-\frac{\beta {\widetilde{v}}_2{\widetilde{x}}}{({\widetilde{x}}+{\widetilde{y}})^2}}&{} ~~~\displaystyle {-\frac{\beta {\widetilde{y}}}{{\widetilde{x}}+{\widetilde{y}}}-c} &{} ~~0\\ \ \\ \displaystyle {-\frac{\beta {\widetilde{v}}_2{\widetilde{y}}}{({\widetilde{x}}+{\widetilde{y}})^2}} &{}~~~\displaystyle {\frac{\beta {\widetilde{v}}_2{\widetilde{x}}}{({\widetilde{x}}+{\widetilde{y}})^2}} &{} ~~\displaystyle {\frac{ \beta {\widetilde{y}}}{{\widetilde{x}}+{\widetilde{y}}}} &{} ~~ -c\\ \end{array} \right] , \end{aligned}$$

and

$$\begin{aligned} \mathbf{B}=\left[ b_{ij}\right] =\left[ \begin{array}{cccc} 0 &{} ~~~0&{} ~~~0 &{} ~~~0\\ \ \\ 0&{} ~~~0 &{} ~~~0 &{} ~~~0\\ \ \\ b_{31} &{}~~~b_{32} &{} ~~~b_{33} &{} ~~~b_{34}\\ \ \\ 0 &{} ~~~0 &{} ~~~0&{} ~~~0\\ \end{array} \right] , \end{aligned}$$

where

$$\begin{aligned}&a_{11}=r\left[ 1-\frac{(2{\widetilde{x}}+{\widetilde{y}})}{k}\right] -\frac{\lambda {\widetilde{y}}{\widetilde{v}}_3}{({\widetilde{x}}+{\widetilde{y}})^2}-\mu ,~~~~ b_{31}=b_{32}=\frac{{\bar{b}}({\widetilde{v}}_2+{\widetilde{v}}_3)^2}{a({\widetilde{x}}+{\widetilde{y}})^2}\exp \left[ -\frac{{\widetilde{v}}_2+{\widetilde{v}}_3}{a({\widetilde{x}}+{\widetilde{y}})}\right] ,\\&b_{33}=b_{34}= {\bar{b}}\left[ 1-\frac{{\widetilde{v}}_2+{\widetilde{v}}_3}{a({\widetilde{x}}+{\widetilde{y}})}\right] \exp \left[ -\frac{{\widetilde{v}}_2+{\widetilde{v}}_3}{a({\widetilde{x}}+{\widetilde{y}})}\right] . \end{aligned}$$

Explicitly, the characteristic equation has the form

11 $$\begin{aligned} \xi ^4+\sigma _1\xi ^3+\sigma _2\xi ^2+\sigma _3\xi +\sigma _4+e^{-\tau \xi }[\eta _1\xi ^3+\eta _2\xi ^2+\eta _3\xi +\eta _4]=0, \end{aligned}$$

where

$$\begin{aligned} \sigma _1= & {} -(a_{11}+a_{22} + a_{33} +a_{44}), \\ \sigma _2= & {} (a_{11}+a_{44})(a_{22} + a_{33})-a_{14}a_{41} -a_{24}a_{42}-a_{12}a_{21} +a_{22} a_{33}+a_{11}a_{44}\\ \sigma _3= & {} a_{33}(a_{12}a_{21} - a_{11}a_{22}) +a_{41}(a_{14}a_{22}-a_{12}a_{24} +a_{14} a_{33})\\&+(a_{11}a_{24}-a_{14}a_{21})a_{42} -(a_{14} a_{31}+a_{24} a_{32})a_{43}\\&+a_{24} a_{33}a_{42} +(a_{12}a_{21}-a_{11}a_{22})a_{44} -(a_{11}+a_{22} )a_{33}a_{44},\\ \sigma _4= & {} (a_{12}a_{24}-a_{14}a_{22}) a_{33}a_{41} +(a_{14}a_{21} -a_{11}a_{24}) a_{33}a_{42} \\&+a_{31}a_{43}(a_{14}a_{22} -a_{12}a_{24} ) +a_{32} a_{43}(a_{11}a_{24}-a_{14}a_{21})\\&+a_{33}a_{44}\left( a_{11}a_{22} -a_{12}a_{21}\right) ,\\ \eta _1= & {} - b_{33}, \quad \eta _2=-b_{34}a_{43} + b_{33}(a_{44}+a_{11} +a_{22}),\\ \eta _3= & {} b_{33}(a_{12}a_{21}+a_{14} a_{41}-a_{11}a_{22} +a_{24} a_{42}) - b_{31}a_{14}a_{43}\\&- b_{32}a_{24}a_{43} +b_{34}(a_{11}a_{43} +a_{22}a_{43})-b_{33}(a_{11} a_{44} -a_{22}a_{44}),\\ \eta _4= & {} b_{33}a_{41}\left( a_{12}a_{24}-a_{14}a_{22}\right) +b_{33}a_{42}(a_{14}a_{21} -a_{11}a_{24}) \\&+b_{31}a_{43}(a_{14}a_{22} -a_{12}a_{24} ) +b_{32} a_{43}(a_{11}a_{24}-a_{14}a_{21})\\&+b_{34} a_{43}(a_{12}a_{21} -a_{11}a_{22} ) +b_{33}a_{44}\left( a_{11}a_{22} -a_{12}a_{21}\right) . \end{aligned}$$

At the steady state $E_1$, the characteristic equation (11) has the roots $-(r-\mu )<0$, $-(\alpha +\mu )<0$, and $-c<0$, with the remaining roots $\xi$ being given by the transcendental equation

12 $$\begin{aligned} -c-\xi +{\bar{b}}e^{-\tau \xi }=0. \end{aligned}$$

For $\tau =0$, the condition for stability, i.e. $Re(\xi )<0$, becomes ${\bar{b}}<c$, which simplifies to $b<c$. Let us *suppose* the condition

13 $$\begin{aligned} {\bar{b}}<c \Longleftrightarrow b e^{-c\tau }<c \end{aligned}$$

also holds for *some* $\tau >0$. To check, whether or not the steady state $E_1$ is stable, we look for roots of equation (12) in the form $\xi =\rho +i\kappa$. Substituting this into (12) and separating real and imaginary parts yield

$$\begin{aligned}&-c-\rho +{\bar{b}}e^{-\tau \rho }\cos (\tau \kappa )=0,\\&-\kappa -{\bar{b}}e^{-\tau \rho }\sin (\tau \kappa )=0. \end{aligned}$$

From the first of these equations, we have

$$\begin{aligned} \rho =-c+{\bar{b}}e^{-\tau \rho }\cos (\tau \kappa ), \end{aligned}$$

and since ${\bar{b}}<c$, this equation has no roots with $\rho >0$, which implies that the steady state $E_1$ is stable. If ${\bar{b}}>c$, due to the definition of ${\bar{b}}$, this means that $b>c$, and hence, the steady state $E_1$ is unstable already at $\tau =0$, though the analysis we have just performed shows that this steady state can get stabilised for sufficiently large $\tau$ that ensures the condition (13) holds.

The disease-free steady state $E_2$ only exists when ${\bar{b}}>c$, i.e. when the plant-only equilibrium $E_1$ is unstable. At the steady state $E_2$, one eigenvalue of the characteristic equation is $-(r-\mu )<0$, and the remaining roots satisfy the following equation

14 $$\begin{aligned} L_1(\xi )\cdot L_2(\xi )=0, \end{aligned}$$

where

$$\begin{aligned}&L_1(\xi )=-\xi -c+c\left( 1-\ln \left( {\bar{b}}/c\right) \right) e^{-\tau \xi },\\&L_2(\xi )={\bar{x}}\xi ^2+(\alpha +c+\mu ){\bar{x}}\xi +c{\bar{x}}(\alpha +\mu )-\lambda \beta {\bar{v}}_{2}. \end{aligned}$$

Looking at the transcendental equation $L_1(\xi )=0$, we note that when $\tau =0$, $\xi =-c\ln \left( {\bar{b}}/c\right) <0$. Since $\xi =0$ is not a root of $L_1(\xi )$, we look at a possibility of $\xi$ crossing the imaginary axis from left to right for some $\tau >0$. For this to happen, we would need $\xi =i\omega$. Substituting this into $L_1(\xi )=0$ and separating real and imaginary parts give

$$\begin{aligned}&-c+c\left( 1-\ln \left[ {\bar{b}}/c\right] \right) \cos (\omega \tau ),\\&\omega =-c\left( 1-\ln \left( {\bar{b}}/c\right] \right) \sin (\omega \tau ). \end{aligned}$$

Since ${\bar{b}}>c$, as required for the feasibility of the steady state $E_2$, the first of these two equations can never be satisfied, and hence, the equation $L_1(\xi )=0$ does not have purely imaginary roots, and all of its roots have a negative real part.

The roots of the quadratic equation $L_2(\xi )=0$ have a negative real part, provided

$$\begin{aligned} c{\bar{x}}(\alpha +\mu )-\lambda \beta {\bar{v}}_{2}<0, \end{aligned}$$

which, in light of the definition (8), can be rewritten as $R_0<1$. When $R_0=1$, $\xi =0$ will be a root of $L_2(\xi )=0$, and for $R_0>1$, one of the two roots of $L_2(\xi )=0$ will be positive.

Hence, we have the following result.

### Theorem 1

Disease-free equilibrium $E_2$ of the model (3) is stable for $R_0<1$, unstable for $R_0>1$, and undergoes a steady-state bifurcation at $R_0=1$.

Looking at stability of the endemic equilibrium $E^*$, for $\tau =0$, the characteristic Eq. (11) reduces to

15 $$\begin{aligned} \xi ^4+\alpha _1\xi ^3+\alpha _2\xi ^2+\alpha _3\xi +\alpha _4=0, \end{aligned}$$

where we have introduced

$$\begin{aligned} \alpha _1=(\sigma _1+\eta _1),\quad \alpha _2=(\sigma _2+\eta _2),\quad \alpha _3=(\sigma _3+\eta _3),\quad \alpha _4=\sigma _4+\eta _4. \end{aligned}$$

The Routh–Hurwitz criterion gives that all roots of this characteristic equation have negative real parts, provided the following conditions hold

16 $$\begin{aligned} \alpha _1>0\quad \alpha _4>0,\quad \alpha _1\alpha _2-\alpha _3>0,\quad (\alpha _1\alpha _2-\alpha _3)\alpha _3-\alpha _1^2\alpha _4>0. \end{aligned}$$

To explore the possibility of endemic equilibrium $E^*$ losing stability via Hopf bifurcation, let us consider $\theta \in {\mathbb {R}}$ to be a generic bifurcation parameter (i.e. one of the many model parameters). The following result can be proven in a manner similar to (Venturino et al. 2016).

### Theorem 2

For $\tau =0$, the endemic equilibrium $E^*$is stable if the conditions (16) hold. At $\theta = \theta ^*$, the steady state $E^*$ undergoes a Hopf bifurcation, if

$$\begin{aligned} \alpha _1(\theta ^*)\alpha _2(\theta ^*)\alpha _3(\theta ^*)-\alpha _3^2(\theta ^*)-\alpha _4(\theta ^*)\alpha _1^2(\theta ^*)=0, \end{aligned}$$

and

$$\begin{aligned} \Big [\alpha _1^3 \alpha '_2\alpha _3(\alpha _1-3\alpha _3)\ne 2(\alpha _2\alpha _1^2-2\alpha _3^2)(\alpha '_3\alpha _1^2-\alpha '_1\alpha _3^3)\Big ]\Big |_{\theta =\theta ^*}\ne 0, \end{aligned}$$

where prime denotes differentiation with respect to $\theta$.

Assuming the Routh–Hurwitz conditions (16) hold, the steady state $E^*$ is stable for $\tau =0$, and the next question is whether it can lose stability as the maturation time delay $\tau$ increases. To answer this question, we refer back to the characteristic Eq. (11). At a critical value of $\tau$ corresponding to a possible stability switch, this characteristic equation will have a pair of complex conjugate eigenvalues with zero real part, which we denote as $\xi = \pm i\zeta$, $\zeta > 0$. Substituting $\xi = i\zeta$ into characteristic Eq. (11), and separating real and imaginary parts, gives

17 $$\begin{aligned}&\zeta ^4-\sigma _2\zeta ^2+\sigma _4=[\zeta ^2\eta _2-\eta _4]\cos {\zeta \tau }-[\zeta \eta _3]\sin {\zeta \tau },\nonumber \\&\sigma _1\zeta ^3-\sigma _3\zeta =[\zeta ^2\eta _2-\eta _4]\sin {\zeta \tau }+[\zeta \eta _3]\cos {\zeta \tau }. \end{aligned}$$

Squaring and adding these two equations yield the following polynomial equation for $\zeta$,

18 $$\begin{aligned} H(z) =z^4+\gamma _1 z^3+\gamma _2 z^2+\gamma _3 z+\gamma _4=0, \quad z=\zeta ^2, \end{aligned}$$

where

$$\begin{aligned}&\gamma _1 =\sigma _1^2-2\sigma _2, \quad \gamma _2 =2\sigma _4+\sigma _2^2-2\sigma _1\sigma _3-\eta _2^2,\\&\gamma _3 =-2\sigma _4 \sigma _2+\sigma _3^2+2\eta _2\eta _4-\eta _3^2,\quad \gamma _4= \sigma _4^2-\eta _4^2. \end{aligned}$$

Since $H(0) = \gamma _4$ and $\lim _{z\rightarrow \infty }H(z)=\infty$, if $\gamma _4<0$, there exists at least one positive root $z\in (0,\infty )$ satisfying Eq. (18), but, of course, there can be more than one such root. If Eq. (18) does have at least one positive root, $\zeta _0$, then the characteristic Eq. (11) will have a pair of purely imaginary roots $\pm i\zeta _0$ for that particular value of the time delay $\tau$. Without loss of generality, let us assume that Eq. (18) has eight positive real roots denoted as $\zeta _1, \zeta _2, \ldots , \zeta _{8}$. For every fixed $\zeta _k$ $(k = 1, 2, \ldots , 8)$, the corresponding critical value of time delay is

$$\begin{aligned}&\displaystyle {\tau _k^{(n)}=\frac{1}{\zeta _k}\arccos \left[ \frac{\zeta _k^2\eta _4[\sigma _1\omega _0^2-\sigma _3]+(\eta _2\zeta _k^2-\eta _4)[\zeta _k^4-\sigma _2\zeta _k^2+\sigma _4]}{(\eta _2\omega _0^2-\eta _4)^2+\eta _3^2\zeta _k^2}+2\pi n\right] ,}\\&\quad n=0,1,2,3,\dots \end{aligned}$$

Let $\tau _0 = \min \{\tau ^{ (j)}_k\}$, $\zeta _0 = \zeta _k|_{\tau =\tau _0},$ $k = 1,\ldots ,8$. Taking the derivative of the characteristic equation (11) with respect to $\tau$ yields

$$\begin{aligned} \left( \frac{\mathrm{d}\xi }{\mathrm{d}\tau }\right) ^{-1}=\frac{4\xi ^3+3\sigma _1\xi ^2+2\sigma _2\xi +\sigma _3}{\xi (\eta _1\xi ^3+\eta _2\xi ^2+\eta _3\xi +\eta _4)}e^{\xi \tau }+\frac{3\eta _1\xi ^2+2\eta _2\xi +\eta _3}{\xi (\eta _1\xi ^3+\eta _2\xi ^2+\eta _3\xi +\eta _4)}-\frac{\tau }{\xi }. \end{aligned}$$

We also have

$$\begin{aligned} \text{ sgn }\left[ \frac{\mathrm{d}~\mathrm{Re} \{\xi (\tau )\}}{\mathrm{d}\tau }\Bigg |_{\tau =\tau _0}\right] =\text{ sgn }\left[ \mathrm{Re} \left( \frac{\mathrm{d}\xi }{\mathrm{d}\tau }\right) \Bigg |_{\xi =i\zeta _0}\right] =\text{ sgn }\left\{ \left[ \mathrm{Re} \left( \frac{\mathrm{d}\xi }{\mathrm{d}\tau }\right) ^{-1}\right] _{\xi =i\zeta _0}\right\} . \end{aligned}$$

Substituting equations (17) into this expression or using the methodology of Li et al. (2011), we find that $dRe(\xi (\tau _0))/d\tau$ and $H'(z_0)$ have the same sign.

### Theorem 3

Suppose the conditions $R_0>1$ and (16) are satisfied. Then, the following results hold.

(i) If Eq. (18) does not have real positive roots (e.g. when all $\gamma _i>0, i=1,2,3,4$), the endemic equilibrium $E^*$ is locally asymptotically stable for all $\tau \ge 0$.

(ii) If Eq. (18) has at least one real positive root $z_0$, and $H'(z_0)\ne 0$, then the endemic equilibrium $E^*$ is locally asymptotically stable for $\tau \in [0, \tau )$, unstable for $\tau >\tau _0$, and undergoes a Hopf bifurcation at $\tau =\tau _0$.

### Remark 1

If the endemic steady state $E^*$ is biologically feasible but the conditions (16) are not satisfied, then this steady state is unstable already for $\tau =0$. In such case, increasing $\tau$ can result in the stabilisation of this steady state following an inverse supercritical Hopf bifurcation.

## Numerical Stability Analysis and Simulations

In this section, we use numerical simulations to further explore the role of different parameters in the system dynamics, and to illustrate different types of dynamical behaviour that can be observed in the model. Since our model is based on the model of Holt et al. (1997), we follow that work to establish baseline values of parameters. Specifically, the death rate *c* of vectors is chosen to have the same value of $c=0.12$, the growth rates of plants are chosen to be at a slightly higher value of $r=0.3$ compared to the range of 0.025–0.2, and the carrying capacity of plants $k=1$ is chosen to be at the higher value of the range 0.01–1 explored in Holt et al. (1997). Vector abundance *a* was explored in Holt et al. (1997) in the range $0-2500$, with the baseline value of $a=500$, and we chose this to be $a=300$, while plant death/roguing rate $\alpha$ is chosen to be $\alpha =0.012$ within the range of 0–0.033 explored in Holt et al. (1997). Finally, the transmission rates $\lambda$ and $\beta$ from vectors to plants, and from plants to vectors, were varied in the range 0–0.06, respectively, 0–0.006, against the range 0.002–0.032 studied in Holt et al. (1997).

**Fig. 1:**
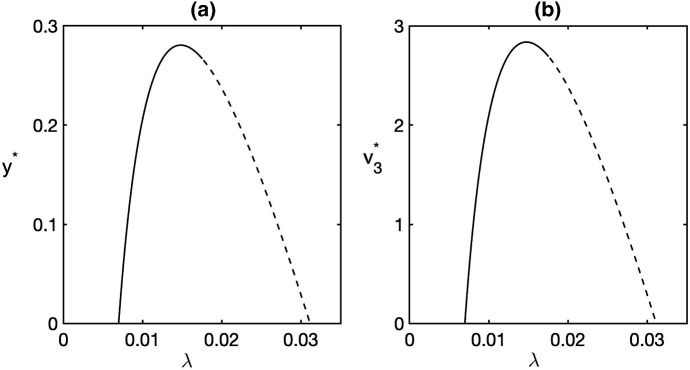
Equilibrium values of infected plants *y* and infectious mature vectors $v_3$ at the endemic steady state $E^*$. Parameter values are as follows $b=0.3$, $r=0.3$, $k=1$, $\beta =0.0045$, $c=0.12$, $\mu =0.06,~\alpha =0.012$, $a=300$, $\tau =0$, and $\lambda$ is varied. Solid (dashed) lines represent stable (unstable) endemic steady state $E^*$

**Fig. 2:**
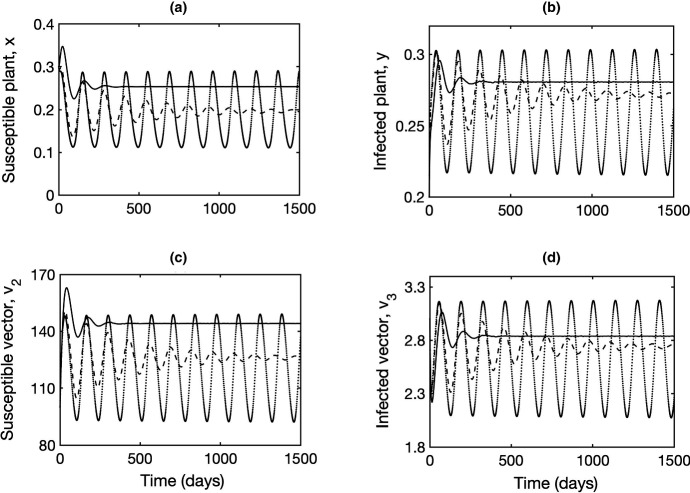
Numerical solutions of the model (3) with parameter values as in Fig. 1, and $\lambda =0.015$ (solid), $\lambda =0.017$ (dashed), $\lambda =0.0175$ (dotted)

Figure 1 shows the dependence of equilibrium values of the numbers of infected plants and mature infective vectors at the endemic steady state depending on the basic reproduction number $R_0$ for $\tau =0$. To produce this figure, we have fixed the values of all parameters and only varied $\lambda$, the rate of disease transmission from vectors to plants. We observe that for $R_0<1$, both these equilibrium values are zero, which corresponds to a situation where either the boundary equilibrium $E_1$ or the disease-free steady state $E_2$ is stable, depending on whether the condition (13) is satisfied. As $R_0$ passes through 1, the disease-free steady state loses its stability, and the endemic steady state $E^*$ becomes biologically feasible, in agreement with Theorem 1 and the condition (7). Further increase in the value of $R_0$ (in the particular case shown in Fig. 1, this corresponds to an increase in the transmission rate $\lambda$) results in the loss of stability of the endemic steady state $E^*$ through a Hopf bifurcation, as described in Theorem 3. Such Hopf bifurcation can lead to the emergence of stable periodic oscillations around the endemic equilibrium, as shown in Fig. 2.

**Fig. 3:**
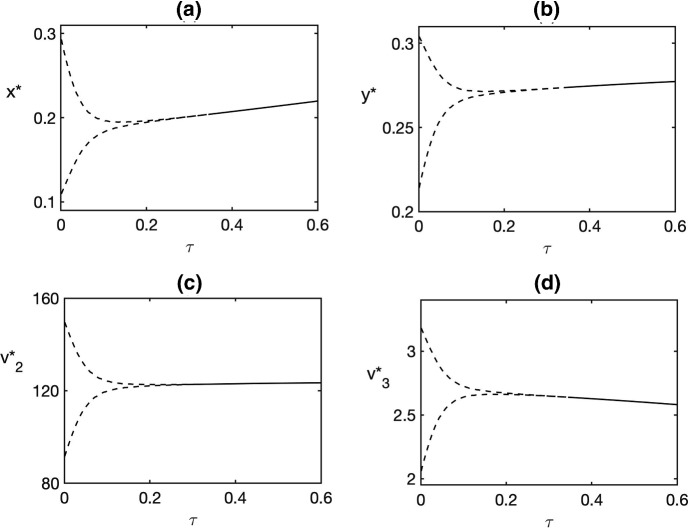
Bifurcation diagram for the endemic equilibrium $E^*$ with maturation time $\tau$ as a bifurcation parameter. The values of parameters are as in Fig. 1, with $\lambda =0.0175$. Solid line indicates a stable steady state, and dashed line indicates minima and maxima of the periodic solution around an unstable steady state

**Fig. 4:**
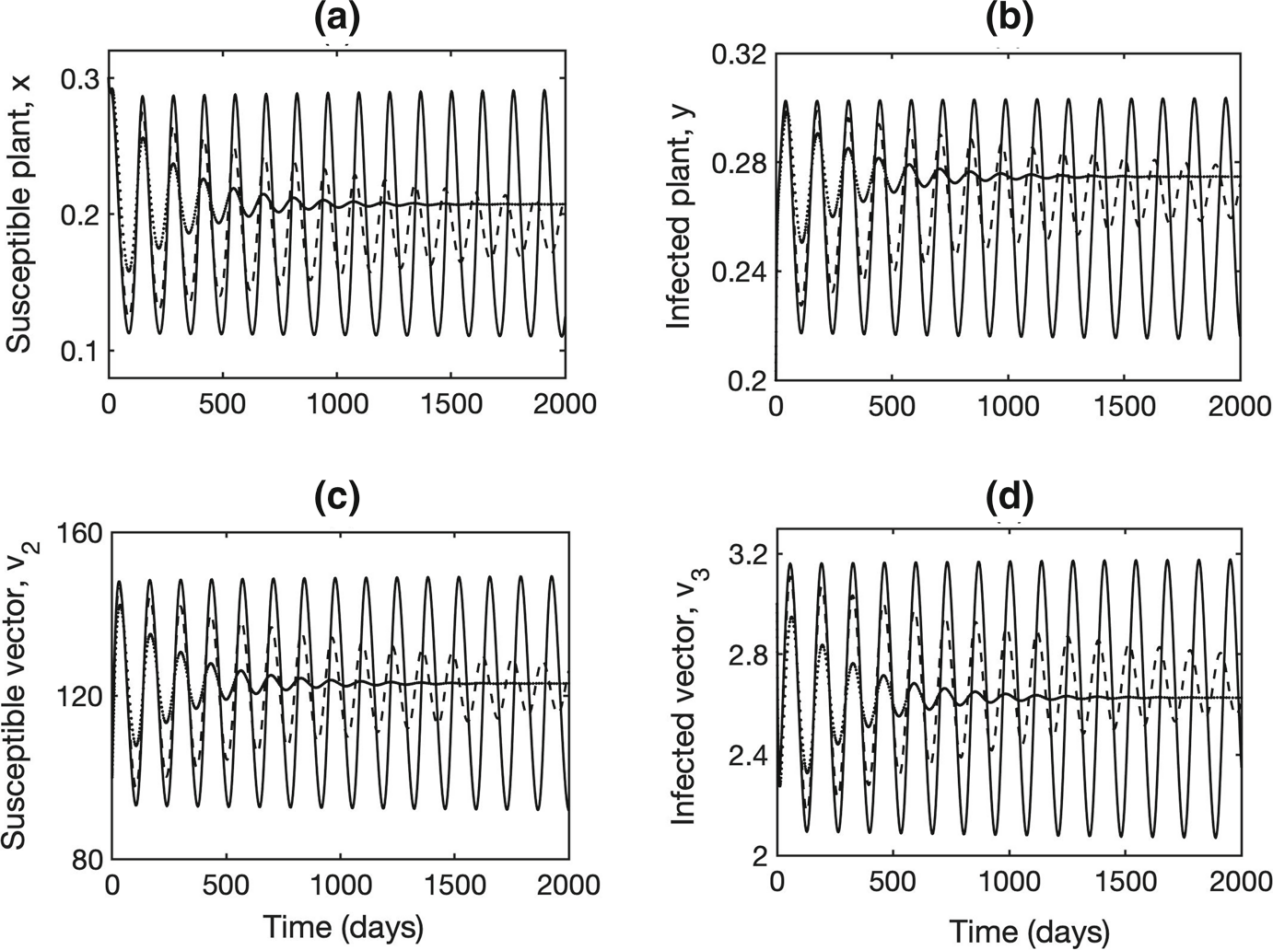
Numerical solutions of the model (3) with parameter values as in Fig. 1, with $\lambda =0.0175$ and $\tau =0$ (solid line), $\tau =0.1$ (dashed line), $\tau =0.4$ (dotted line)

**Fig. 5:**
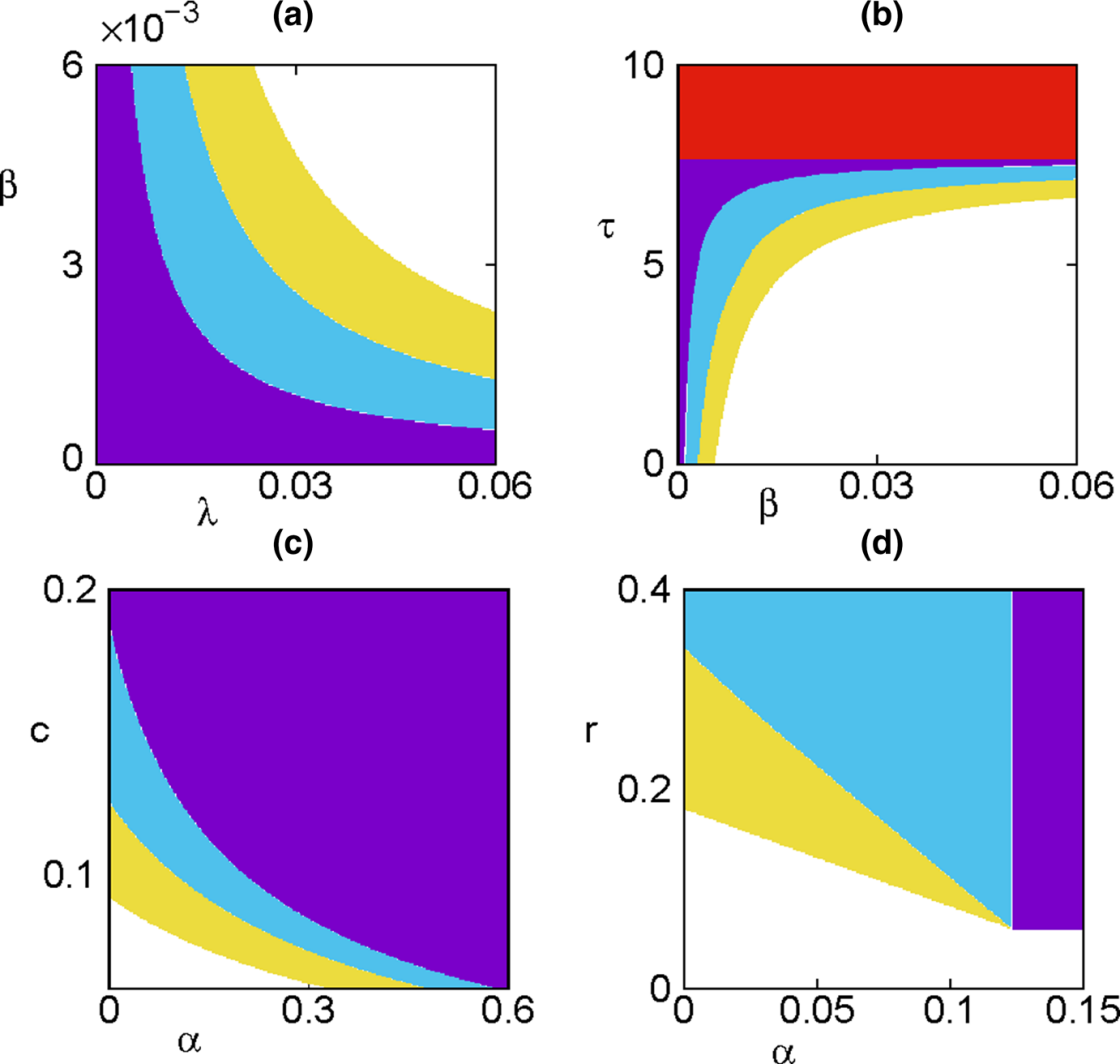
(Colour figure online) Regions of stability of different dynamical states with parameter values as in Fig. 1. Axial equilibrium $E_1$ is stable in the red region, disease-free steady state $E_2$ is stable in the purple region, and endemic equilibrium $E^*$ is stable in the light-blue region and unstable in yellow region, where a periodic solution around $E^*$ is stable. White region corresponds to a state (0, 0, 0, 0) of complete extinction

Figure 3 illustrates a case corresponding to Remark 1, where for $\tau =0$, the endemic steady state $E^*$ is unstable, and the system exhibits stable periodic oscillations around this equilibrium. As the time delay $\tau$, increases, this steady state undergoes an inverse supercritical Hopf bifurcation, which results in disappearance of periodic solutions and stabilisation of $E^*$. Numerical solutions of the model (3) during such transition are shown in Fig. 4.

Figure 5 demonstrates how stability of different steady states depends on system parameters,with characteristic eigenvalues computed using traceDDE (Breda et al. 2006) In plot (a), we observe that when the product of transmission rate $\lambda$ from vectors to plants and $\beta$ from plants to vectors is small, the disease-free steady state $E_2$ is stable, and then, as the value of this product increases, the disease-free steady state loses its stability, giving rise to endemic steady state $E^*$. This is perfectly consistent with condition (7) and the expression (8) for the basic reproduction number, which is proportional to that product of transmission rates. Further increase of $\lambda \beta$ leads to a loss of stability of the endemic steady state through a Hopf bifurcation, and eventually, this steady state becomes biologically infeasible, once $\lambda$ exceeds an upper bound as determined by the existence condition for that steady state (7). Figure 5b explores bifurcation dynamics depending on the rate $\beta$ of disease transmission from plants to vectors, and the vector maturation period $\tau$. We notice that unlike an earlier situation, now for a sufficiently large maturation period, the system will approach a stable plant-only steady state $E_1$, which will happen for $\tau >\tau _c=[\log (b/c)]/c$, in accordance with stability condition (13). Biologically, this represents a scenario where the spread of infection is reduced due to a long time that it takes for vector to mature (since infection is only spread by and to mature vectors). As a result, the infection is not spread fast enough to be maintained in the population of plants and vectors, and the vectors themselves are not replenished sufficiently quickly, and hence, they go extinct, and the system settles on a plant-only steady state. For values of $\tau <\tau _c$, we again observe a disease-free steady state for very small values of disease transmission rate, and as this rate increases, the same sequence of transitions to a stable endemic state and a periodic solution around that state takes place. For higher still values of the disease transmission rate, the system again is not able to maintain the plant and vector populations due to a high infection rate, and as a result, both of those populations go extinct.

Figure 5c shows that for sufficiently high disease-induced death rate $\alpha$ of infected plants or their removal by roguing, only the disease-free steady state $E_2$ is stable, regardless of the death rate *c* of adult vectors. If infected plants are not removed so quickly, i.e. for lower values of $\alpha$, one again observes a sequence of transitions from a periodic solution around the endemic steady state $E^*$ to a stable steady state $E^*$, followed by the stable disease-free steady state $E_2$. This is explained by the fact that as adult vectors are dying faster (possibly, due to the use of pesticides) compared to how they are reproducing, this reduces the overall disease spread and ultimately results in disease eradication. For very small death rates of infected plants and vectors, the disease overwhelms both populations, resulting in their extinction. When looking at the relative influence of the plant growth rate *r* and the death/removal rate $\alpha$ of infected plants, we observe that for sufficiently small values of *r*, the population of plants is not replenished fast enough, and as a result, both plant and vector populations go extinct. For sufficiently small values of $\alpha$, we observe the transition from extinction steady state to a periodic solution around the endemic steady state, followed by the stable endemic steady state for increasing values of the plant growth rate *r*. This is an interesting observation, which suggests that effectively, increasing the rate at which plants are replenished, possibly through the use of nutrients/fertilisers, also inadvertently facilitates the maintenance of infection. With the value of basic reproduction number $R_0$ being independent of *r*, there is some minimum value of $\alpha$ as determined by the expression (8) and Theorem 1, beyond which the disease-free steady state is stable, whenever it exists.

## Discussion

In this paper, we have studied the dynamics of a vector-borne cassava mosaic disease of plants, with particular emphasis on the role played by maturation time of vectors. One particular novelty of the model is the delayed ratio dependence (vector density relative to host density) driving the fecundity of vectors. Stability analysis has revealed that the basic reproduction number increases with disease transmission rates and decreases with maturation delay, so the disease-free steady state is stable, provided the vector mature sufficiently slowly to prevent further spread of infection. For a very large maturation delay, the vector population can no longer be maintained, and the system only has a stable boundary equilibrium with healthy plants only.

We have obtained conditions for existence and stability of the plant-only, disease-free and endemic equilibria and have explored how the behaviour of the model depends on its parameters, with particular emphasis on the role on maturation delay of vectors. Larger disease transmission rates lead to a transition from a stable disease-free steady state to a stable endemic equilibrium, which then loses stability through a Hopf bifurcation, giving rise to periodic solution, and the same transitions are observed for smaller maturation delays. Biological interpretation of this observation could be that a lower maturation delay means a larger pool of mature susceptible vectors that can become carriers of the disease, and with the Cassava mosaic disease being caused by a persistently circulating virus, such vectors would then be infected for the duration of their lifetime, resulting in a bigger contribution to the force of infection. Similarly, in order for infection to be maintained in the combined plant-vector system, there should be sufficient time for it to be passed from vectors to plants, and from plants to vectors. Hence, the endemic steady state is only biologically feasible when the death rate of infected plants is not too high, and when the the rate of disease transmission is not too low. Interestingly, increasing disease transmission rate(s) actually results in a destabilisation of the endemic steady state and the emergence of stable periodic oscillations around this state. Of course, in a real biological situation, maturation delay of vectors cannot be changed. However, since feasibility and stability of different steady states are determined by a number of different parameters, controlling other characteristics, such as disease transmission or plant growth rates via pesticides and/or nutrients and fertilisers can result in control and eradication of the Cassava mosaic disease, and the results presented in this paper provide insights into parameter regimes where this is possible to achieve.

There are several directions, in which the work presented in this paper can be extended. One possibility is to make the model more realistic by including a time delay associated with disease development in plants and vectors, i.e. disease latency. This would result in a multi-delay model, making the analysis more challenging. However, it would make the model more realistic in terms of carefully accounting for different characteristic time scales, some of which can be of the same order, which can potentially result in interesting and complex dynamics. Another potential avenue is to model maturation and latency using distributed time delays, thus accounting for the fact that in reality neither of the processes of maturation and/or disease transmission happens after exactly the same fixed period of time for all plants and all vectors. One could then use available experimental data to infer possible distributions of maturation and latency periods and use those distributions for analysis and simulations.
